# Supplementary material for: Three-dimensional simulation of the pancreatic parenchyma, pancreatic duct and vascular arrangement in pancreatic surgery using a deep learning algorithm
Source: PLoS One. 2022 Oct 28;17(10):e0276600. doi: 10.1371/journal.pone.0276600 (PMC9616217; doi:10.1371/journal.pone.0276600)
Supplement: Attachment — Submitted filename: Respond_to_reviewer_3DAI.docx [file pone.0276600.s003.docx]

**RESPONSES TO THE REVIEWERS**

October 1, 2022

Dear Editor,

**Title:** Three-dimensional simulation of the pancreatic parenchyma, pancreatic duct and vascular arrangement in pancreatic surgery using a deep learning algorithm

**Authors:** Ryoichi Miyamoto, Amane Takahashi, Aya Ogasawara, Toshiro Ogura, Kei Kitamura, Hiroyuki Ishida, Shinichi Matsudaira, Satoshi Nozu, Yoshiyuki Kawashima

**Name of Journal:** *PLOS ONE*

**Manuscript NO:** PONE-D-22-19378

The manuscript has been revised according to the reviewers’ suggestions. The modified parts are highlighted in red font. Please see the highlighted sentences in the annotated manuscript file. Thank you.

**Reviewer Comments**:

Reviewer #1:

*1.This study focused on the reconstruction of the main pancreatic duct and the blood vessels around the pancreas but did not discuss the value of this model for evaluating the relationship between blood vessels and tumors, such as whether the tumor invades blood vessels, and how effective is its evaluation? Does this model have advantages over CT? This may be a question of greater interest to surgeons.*

We thank the reviewer for this comment. In accordance with the reviewer’s comment, it is also important to evaluate the relationship between blood vessels and tumors in pancreatic surgery. Pancreatic tumors, including pancreatic cancer, have an unclear contrast enhancement effect using dynamic MDCT images. Semiautomatic image extraction of pancreatic tumors is very difficult. However, by performing anatomical simulation using a 3D image including a manually masked tumor area, it is possible to grasp the positional relationship between the tumor position and the blood vessel in a three-dimensional view and to plan the surgical method, including consideration of the blood vessel to be resected together.

Furthermore, the surgical team was able to view the preoperative 3D image on a large display during the actual surgery, enabling discussion of the critical points of the surgical procedure. We assumed that these findings have a great advantage over MDCT.

To achieve the function of automatically extracting the tumor area, continued accumulation of AI learning data is needed.

*2. In the Discussion section, the authors should focus more on discussing the clinical benefits and potential applications of the model rather than simply presenting reconstructed data.*

We thank the reviewer for this comment. In accordance with the reviewer’s comment, we revised the manuscript on Page 14, Lines 23 to Page 15, Lines 12.

In terms of the clinical benefits and potential applications, we assumed that sharing of the anatomical images with the surgical staff contributed to the reduced intraoperative blood loss in conjunction with the mastery of surgical techniques, advances in surgical instruments and perioperative management in pancreatic surgery.

Another clinical benefit of creating preoperative 3D images was assumed to be evaluating the residual pancreatic volume. Our previous studies have already reported that postoperative pancreatic volume was closely associated with postoperative pancreatic endocrine insufficiency or useful value for predicting postoperative long-term outcomes in pancreatic cancer patients. Therefore, the early identification of these high-risk patients can increase the accuracy of the perioperative benefit/risk assessment and the information given to patients. We suggest that these patients should be more carefully monitored for the presence of postoperative pancreatic endocrine insufficiency or the recurrence of pancreatic cancer during the postoperative follow-up period.

Reviewer #2:

*“the Dice coefficients of pancreatic parenchyma and pancreatic duct extraction were 0.83/0.86 and 0.84/0.77". P values should be added.*

We thank the reviewer for this comment. In accordance with the reviewer’s comment, we revised the manuscript on Page 10, Lines 8, 22, 23 and Page 11, Lines 5.

We sincerely appreciate the reviewers’ comments, which have helped us improve the paper and provided new insights for our study. We hope that the revised manuscript is now suitable for publication in *PLOS ONE*.

Ryoichi Miyamoto

Department of Gastroenterological Surgery, Saitama Cancer Center, 780 Komuro, Ina-machi, Kita-Adachi-gun, Saitama 362-0806, Japan

Phone: +81-48-722-1111; Fax: +81-48-722-1129

Email: goodfirst883@gmail.com
